# The INTERGROWTH-21^st^ Project Neurodevelopment Package: A Novel Method for the Multi-Dimensional Assessment of Neurodevelopment in Pre-School Age Children

**DOI:** 10.1371/journal.pone.0113360

**Published:** 2014-11-25

**Authors:** Michelle Fernandes, Alan Stein, Charles R. Newton, Leila Cheikh-Ismail, Michael Kihara, Katharina Wulff, Enrique de León Quintana, Luis Aranzeta, Aureli Soria-Frisch, Javier Acedo, David Ibanez, Amina Abubakar, Francesca Giuliani, Tamsin Lewis, Stephen Kennedy, Jose Villar, for the International Fetal and Newborn Growth Consortium for the 21st Century (INTERGROWTH-21st)

**Affiliations:** 1 Nuffield Department of Obstetrics & Gynaecology, and Oxford Maternal & Perinatal Health Institute, Green Templeton College, University of Oxford, Oxford, United Kingdom; 2 Department of Psychiatry, Warneford Hospital, University of Oxford, Oxford, United Kingdom; 3 Department of Psychology, United States International University, Nairobi, Kenya; 4 Nuffield Department of Clinical Neurosciences, John Radcliffe Hospital, University of Oxford, Oxford, United Kingdom; 5 Centro de Tecnología e Innovación, Mexico City, Mexico; 6 Neuroscience Business Unit, Starlab Barcelona, SL, Barcelona, Spain; 7 Department of Psychology, Lancaster University, Lancaster, United Kingdom; 8 KEMRI/Wellcome Trust Research Programme, Kilifi, Kenya; 9 Dipartimento di Scienze Pediatriche e dell' Adolescenza, SCDU Neonatologia, Università di Torino, Turin, Italy; Central South University, China

## Abstract

**Background:**

The International Fetal and Newborn Growth Consortium for the 21^st^ Century (INTERGROWTH-21^st^) Project is a population-based, longitudinal study describing early growth and development in an optimally healthy cohort of 4607 mothers and newborns. At 24 months, children are assessed for neurodevelopmental outcomes with the INTERGROWTH-21^st^ Neurodevelopment Package. This paper describes neurodevelopment tools for preschoolers and the systematic approach leading to the development of the Package.

**Methods:**

An advisory panel shortlisted project-specific criteria (such as multi-dimensional assessments and suitability for international populations) to be fulfilled by a neurodevelopment instrument. A literature review of well-established tools for preschoolers revealed 47 candidates, none of which fulfilled all the project's criteria. A multi-dimensional assessment was, therefore, compiled using a package-based approach by: (i) categorizing desired outcomes into domains, (ii) devising domain-specific criteria for tool selection, and (iii) selecting the most appropriate measure for each domain.

**Results:**

The Package measures vision (Cardiff tests); cortical auditory processing (auditory evoked potentials to a novelty oddball paradigm); and cognition, language skills, behavior, motor skills and attention (the INTERGROWTH-21^st^ Neurodevelopment Assessment) in 35–45 minutes. Sleep-wake patterns (actigraphy) are also assessed. Tablet-based applications with integrated quality checks and automated, wireless electroencephalography make the Package easy to administer in the field by non-specialist staff. The Package is in use in Brazil, India, Italy, Kenya and the United Kingdom.

**Conclusions:**

The INTERGROWTH-21^st^ Neurodevelopment Package is a multi-dimensional instrument measuring early child development (ECD). Its developmental approach may be useful to those involved in large-scale ECD research and surveillance efforts.

## Introduction

**Figure 1:**
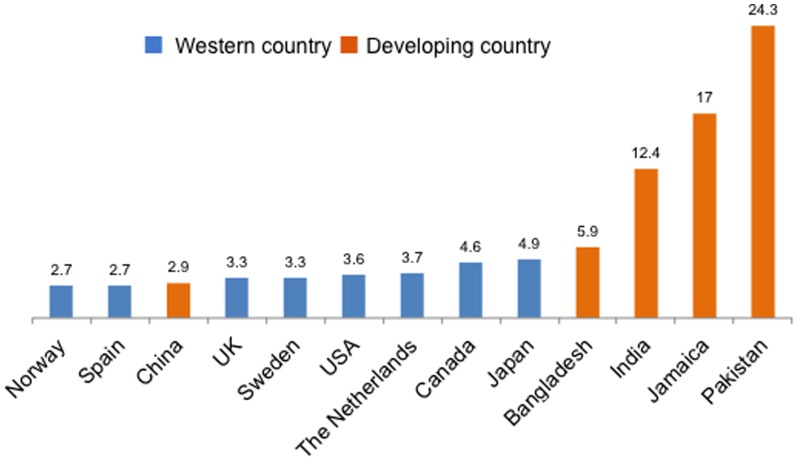
Distribution of the prevalence of severe neurodevelopmental impairment per 1,000 children in select countries. Distribution of prevalence of severe neurodevelopmental impairment per 1,000 children in select countries. Taken from Durkin, The Epidemiology of Developmental Disabilities in Low-Income Countries, Mental Retardation and Developmental Disabilities Research Reviews p. 207 (2002) [2].

Approximately one in ten children have impairments in neurodevelopment, which manifest as disturbances in cognition, auditory processing, vision, behavior, language, attention, motor skills and sleep [1]. Research into the epidemiology of childhood neurodisability has revealed three consistent global trends. First, the prevalence of childhood neurodisability varies widely across geographical locations with higher prevalences reported from low- and middle-income countries (LMICs; 5.3 to 24.3 per 1,000 children) than high-income countries (HICs; 2.0 to 4.5 per 1,000 children) [2]. Second, the reported prevalence of mild levels of neurodisability is consistently higher than that of severe neurodisability in both LMICs and HICs [2]. Third, the prevalence of childhood neurodisability in LMICs is much more variable than in HICs. Among HICs, rates of severe childhood neurodisability range between 2 and 5 per 1,000 children; in LMICs they range from 5.9 per 1,000 in Dhaka, Bangladesh to 24.3 per 1,000 in Karachi, Pakistan [2] (Fig. 1).

A number of genetic, biological and environmental risk factors, operating in the pre- or postnatal period, or both, have been proposed [3]. These are summarized in Table 1. More often than not, these factors do not act in isolation but interact with each other resulting in a multi-factorial etiological matrix in which some, but not all, risk factors may be modifiable. Irrespective of its precise etiological determinants, childhood neurodisability is one of the most important precursors of psychopathology, poor social functioning and educational disadvantage in later life [3], [4]. Attention and behavior problems in early childhood have been associated with higher rates of oppositional/defiant behavior, conduct problems, personality disorder, depression, antisocial lifestyles and suicide attempts in adolescence and adulthood [4]–[7]. Longitudinal studies have also found children with neurodisability to be less likely to be living independently, in paid employment and have cohabitating relationships as adults compared to controls [8]. These effects are most pronounced in those born extremely preterm, those with very low birth weights (<1,000 g) and those with severe neurodevelopmental impairments manifesting early in life [9], [10].

**Table 1:** Summary of major risk factors for early childhood neurodisability.

	Major Risk factors for Early Childhood Neurodisability		
	Genetic Factors	Biological Factors	Environmental Factors
**Prenatal Period**	• Chromosomal disorders	• Poor intra-uterine growth	• Exposure to teratogens, environmental toxins, and substances
	• Gene disorders	• Maternal infections	
			• Maternal exposure to abuse, conflicts & famines
		• Intrauterine infections	
			• Maternal depression & anxiety
**Birth Period**	-	• Low birth weight	• Birth trauma
		• Preterm birth	
		• Birth asphyxia	
**Postnatal Period**	• Metabolic disorders	• Under nutrition	• Exposure to environmental toxins
		• Vitamin deficiencies	• Parental depression& mental health problems
	• Congenital disorders		
		• Infections	• Parenting behavior
	• Malignancies		• Child abuse and neglect
			• Food insecurity & famines
			• Conflicts
			• Natural disasters

Despite the long-term significance of childhood neurodisability and benefit of early diagnosis and treatment in young children, there is limited scientific and clinical agreement on two issues central to the topic: (i) international standards of optimal early child development (ECD) and (ii) standardized, internationally applicable tools to measure these outcomes in young children [11], [12]. Although a sizable body of literature on ECD and childhood neurodisability exists, there is limited comparability among the many studies because of the heterogeneity in methods employed in assessments, the wide ranges of ages assessed, and the focus on specific domains of neurodevelopment rather than global outcomes [11], [12]. Two important reasons for this limited comparability among studies are the lack of: (i) a standardized, robust methodology that is easy and rapid to administer in the field, and that uses a combination of psychological, clinical and neurophysiological tests to measure multiple dimensions of neurodevelopment, and (ii) large, international, prospective cohorts on whom information about potential risk and protective factors for ECD (such as fetal growth, home environment, maternal health and early nutrition) is available.

**Figure 2:**
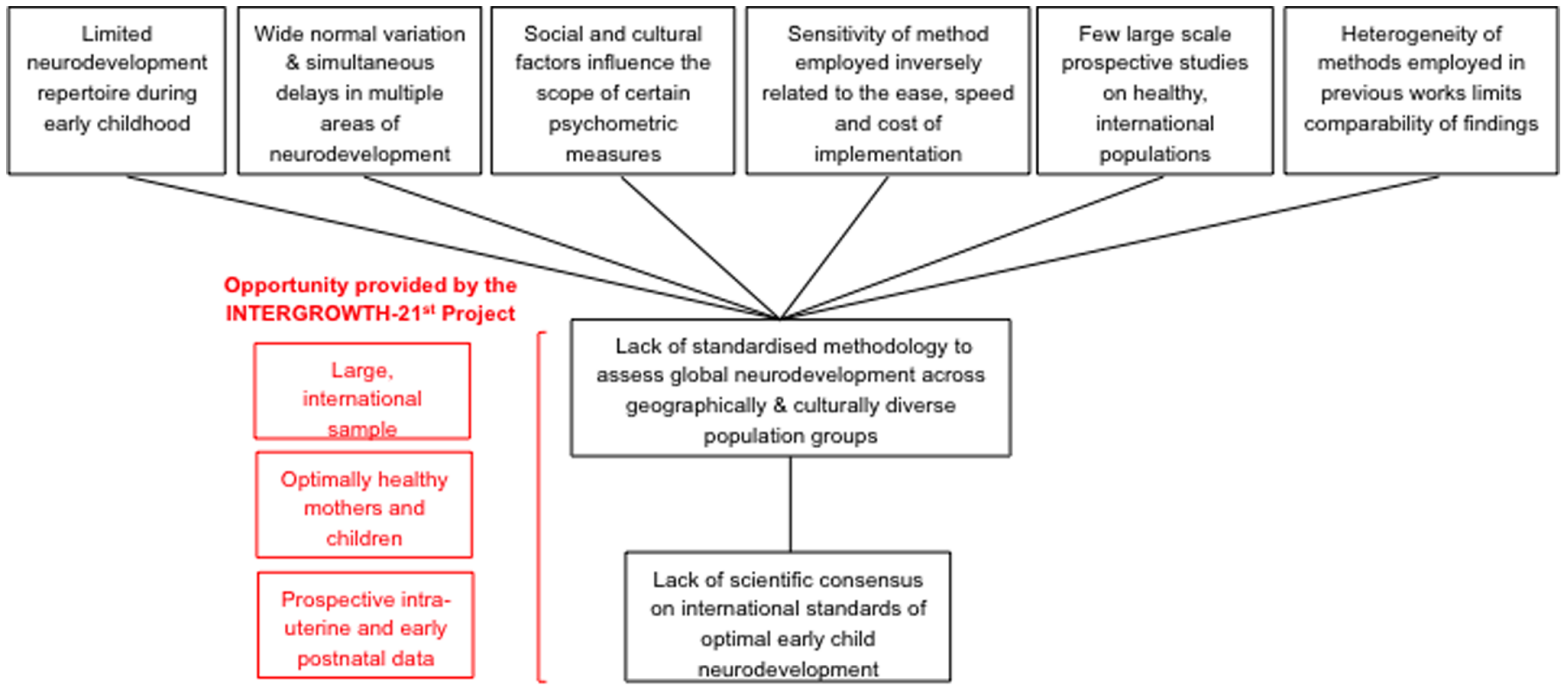
Conceptual and methodological challenges in the assessment of early child development in the context of the INTERGROWTH-21^st^ Project. Conceptual and methodological challenges in the multi-dimensional assessment of early childhood neurodevelopment across low-, middle- and high-income settings (black boxes) and the unique opportunity provided by the INTERGROWTH-21^st^ Project to explore some of these issues (red boxes).

In the context of ECD research, it is important to highlight the many conceptual and methodological challenges in investigating the epidemiology of neurodevelopmental disturbances in young children in a large, international cohort (Fig. 2). First, little is known about childhood neurodisability in LMICs despite the high prevalence of risk factors. In addition, the children most at risk in these settings are unlikely to have been assessed and, therefore, may not be represented in prevalence estimates [2]. Second, although severe disorders may be recognized during infancy, it is difficult to reliably diagnose impairments in speech, cognition or behavior before 3 to 4 years of age [2]. Nevertheless, there is increasing evidence that the earlier the identification and treatment of childhood neurodisability, the better the opportunity for developmental change [2]. Third, the wide normal variation in neurodevelopment among children, simultaneous delays in multiple areas of development, and the logistic implications of carrying out long-term surveillance makes the selection and implementation of an assessment complicated [13]. Fourth, assessments that yield information of clinical prognostic importance are often lengthy, require specialist training and contain culture-specific items making them difficult to implement in the field, especially in international settings [2], [13]. Fifth, studies employing neuroimaging and neurophysiological measurements (e.g. electroencephalography, magnetoencephalography, functional magnetic resonance imaging and eye tracking), although free from cultural biases and of sufficient sensitivity to measure subtle differences in neurodevelopment, are often limited to one domain, are resource intensive and are extremely difficult to implement in the field and in very young children.

### Background: The INTERGROWTH-21^st^ Project

INTERGROWTH-21^st^ (The International Fetal and Newborn Growth Consortium for the 21^st^ Century) is a multicenter, multiethnic, population-based project, conducted between 2008 and 2013, in eight study sites: the cities of Pelotas (Brazil), Turin (Italy), Muscat (Oman), Oxford (UK) and Seattle (USA); Shunyi County, Beijing (China); the central area of Nagpur (India), and the Parklands suburb of Nairobi (Kenya) [14]. Its primary aim was to study growth, health, nutrition and neurodevelopment from <14^+0^ weeks of gestation to 2 years of age, using the same conceptual framework as the WHO Multicentre Growth Reference Study [15], so as to produce prescriptive, international growth standards and a new phenotypic classification of the intrauterine growth restriction and preterm birth syndromes.

The women whose babies contributed to the construction of the international standards describing optimal fetal and newborn growth were selected on the basis of two factors: they were living in environments where the exposure to risk factors known to affect fetal growth was minimal, and they themselves were healthy, non-obese and similarly free of any factors or conditions affecting fetal growth. The study methodology has been published elsewhere in detail [14]. In brief, clinical, nutrition and anthropometric outcomes were measured in mothers and children during pregnancy and at birth, during the first year of life, and at 24 months after birth. The inclusion and exclusion criteria, study protocol, ultrasound manual and other documents are available at www.intergrowth21.org.uk. The recruitment of a large, optimally healthy, international, population-based sample; the use of robust, standardized procedures in all assessments (ultrasound, clinical and anthropometric) including a system of random evaluation and repetition to achieve quality control, and the use of a centralized data management system make the INTERGROWTH-21^st^ birth cohort distinct in its methodology and scope.

In an extension to the 24 month follow-up of the INTERGROWTH-21^st^ Project, an assessment of early child neurodevelopment was included based on the rationale that the Project provided a unique opportunity to profile neurodevelopment outcomes in an optimally healthy sample of children whose nutritional and health needs had been fully met during the first 1,000 days of life (Fig. 2). It is envisioned that the data from this study will add to the current understanding of neurodevelopment trajectories during early childhood by comparing different neurodevelopment functions across a range of contexts and settings as well as relating these functions to intrauterine growth. The Project also provided an opportunity to develop and test a novel, mixed methodology, global approach to the assessment of early child development using objective, robust techniques, which are quick and easy to administer in both high- and low-resource settings by non-professional staff.

Towards this end, a multi-dimensional, mixed methodology, screening package to assess neurodevelopment in international populations of preschoolers was developed. The Package was designed to be easy to administer in the field, in 35 to 45 minutes, by non-specialist research staff. In keeping with the methodology and scope of the INTERGROWTH-21^st^ Project, the Package was designed to measure neurodevelopment in children in an ‘optimally healthy' cohort in accordance with the methodological concepts of standardization, integrated quality assessments and centralized data management. The Package is in use in study sites in Brazil, Kenya, India, Italy and the UK where more than 700 children have been assessed to date. The objectives of this paper are to: (1) outline the methodology leading to the development of the Package and the challenges involved in this process and (2) describe the characteristics of the INTERGROWTH-21^st^ Neurodevelopment Package.

## Materials and Methods

### Study sites and participants

Five INTERGROWTH-21^st^ Project sites are implementing the INTERGROWTH-21^st^ Neurodevelopment Package. These are the Universidade Federal de Pelotas, Pelotas, Brazil; Aga Khan University Hospital, Nairobi, Kenya; Ketkar Hospital, Nagpur, India; Universita' di Torino, Turin, Italy; and the John Radcliffe Hospital, Oxford, UK. A total of 4607 optimally healthy mothers were recruited from these sites [16], [17], their characteristics are presented in Table 2 [17].

**Table 2:** Inclusion criteria to be met by mothers at booking for recruitment in the INTERGROWTH-21^st^ Project.

Inclusion criteria for the INTERGROWTH-21^st^ Project
**Maternal Characteristics:**
a) Age ≥18 and <35 years
b) Body mass index ≥18.5 and <30 kg/m2
c) Height ≥153 cm
d) Singleton pregnancy
e) A known date of the last menstrual period with regular cycles (defined as 28 days ±4 days) without hormonal contraceptive use, or breastfeeding in the 2 months before pregnancy
f) Natural conception
g) No relevant past medical history (refer to screening form), with no need for long term medication (including fertility treatment and over-the-counter medicines, but excluding routine iron, folate, calcium, iodine or multivitamin supplements)
h) No evidence of socio-economic constraints likely to impede fetal growth identified using local definitions of social risk
i) No use of tobacco or recreational drugs such as cannabis in the 3 months before or after becoming pregnant
j) No heavy alcohol use (defined as >5 units (50 ml pure alcohol) per week) since becoming pregnant
k) No more than one miscarriage in the 2 previous consecutive pregnancies
l) No previous baby delivered pre-term (<37 weeks) or with a birth weight <2500 g or >4500 g
m) No previous neonatal or fetal death, previous baby with any congenital malformations, and no evidence in present pregnancy of congenital disease or fetal anomaly
n) No previous pregnancy affected by pre-eclampsia/eclampsia, HELLP (Hemolysis, elevated liver enzymes & low platelets) syndrome or a related pregnancy-associated condition
o) No clinically significant atypical red cell alloantibodies
p) Negative urinalysis
q) Systolic blood pressure <140 mmHg and diastolic blood pressure <90 mmHg
r) No diagnosis or treatment for anemia during this pregnancy
s) No clinical evidence of any other sexually transmitted diseases, including syphilis and clinical Trichomoniasis
t) Not in an occupation with risk of exposure to chemicals or toxic substances, or very physically demanding activity to be evaluated by local standards. Also women should not be conducting vigorous or contact sports, as well as scuba diving or similar activities

### Ethics

Participants in the INTERGROWTH-21^st^ Project were informed about the study procedures, and voluntarily consented to participation. Written consent was obtained from all adult participants. Parents/guardians provided written informed consent on behalf of their children enrolled in the Project. Participants (or their parents/guardians in the case of children) provided written consent for their clinical records to be used in the Project. The INTERGROWTH-21^st^ Project was approved by the Universidade Federal de Pelotas, Faculdade de Medicina comitê de ética em pesquisa (Ref: OF.051/09), Beijing Obstetric and Gynaecology Hospital, Capital Medical University; the Indian Council of Medical Research, the Indian Ministry of Health and Family Welfare and the Institutional Ethics Committee, Ketkar Hospital, Nagpur (Ref: 5/7/314/2008-RHN); Servizio Sanitario Nazionale – Regione Piemonte, Aziende Ospedaliere OIRM/S.Anna, Oridine Mauriziano di Torino, Comitato Etico Interaziendale (Ref: G9947/CEI/C.27.2); the Aga Khan University Health Research Ethics Committee (Ref: AKU-09-106), the Oman Research and Clinical Studies Committee (Ref: MH/DGHA/DNCD/R&C/47/09), the Oxfordshire Research Ethics Committee ‘C’, United Kingdom (Ref: 08/H0606/139) and the University of Washington, Human Subjects Division, Human Subjects Review Committee (Ref: 36341).

### Conceptual issues guiding the INTERGROWTH-21^st^ Project Neurodevelopment Assessment

Figure 2 illustrates the conceptual framework behind the rationale to include an assessment of neurodevelopment in the INTERGROWTH-21^st^ Project. Prior to the selection of a neurodevelopment measure for the Project, an Infant Development Group consisting of international experts in pediatric medicine, pediatric neurology, child development, child psychology, neuroscience, electroencephalography, vision, obstetric medicine and epidemiology was constituted. The Group's remit was to review the cohort's characteristics and the requirements of the Project so as to develop a set of criteria for a neurodevelopment measure to meet the Project's needs (see Table 3). As a preliminary step, the Group identified a number of conceptual, methodological and logistical challenges to the selection, design or development of a neurodevelopment measure in a large, multi-site study of young children. These are listed in Table 4.

**Table 3:** Criteria to be met by a neurodevelopment measure for inclusion in the INTERGROWTH-21^st^ Project.

INTERGROWTH-21^st^ Project criteria for a neurodevelopment measure
**Essential criteria:**
i. The measure must be suitable for assessing neurodevelopment in children at 2 years of age.
ii. The measure must be sufficiently sensitive to detect subtle differences in neurodevelopment in a largely healthy cohort of children
iii. The measure must characterize neurodevelopmental outcomes in children across a spectrum ranging from normal to mild, moderate and severe disturbances, and not assess severe neurodevelopmental delay only.
iv. The measure must assess neurodevelopmental outcomes with the highest possible degree of objectivity to minimize rate, reporter and cultural biases.
v. The measure must be suitable for use in HICs as well as LMICs, and must not contain items that are culture-specific.
vi. The measure must assess multiple domains of neurodevelopment, including motor development, cognition, language skills and behavior.
vii. The total duration of assessment for each individual child must not exceed 50 minutes.
viii. It must be easy to train local field workers to administer the tool and no specialist training in psychiatry, psychology or related disciplines should be necessary.
**Desirable criteria:**
i. The tool should characterize the child's performance on each item on a Likert-like scale yielding a range of possible outcomes for each item rather than binary categorization of the child's performance as pass or fail.
ii. The tool should include a combination of methodologies for assessing infant neurodevelopment including direct tests, parent reports and/or neurophysiological methods.
iii. If devised and tested in low resource settings in LMICs the tool should be appropriate for use in high-income settings in the HICs as well as LMICs.

**Table 4:** Challenges to the selection, design and development of a neurodevelopment assessment for use in young children and in multiple international settings in the INTERGROWTH-21^st^ Project.

Challenges in selection of a measure of early child development for the INTERGROWTH-21^st^ Project	
**Conceptual**	A range of neurodevelopmental outcomes should be measured to assess simultaneous delays in multiple areas of neurodevelopment
	Careful selection of an assessment tool sensitive enough to detect subtle differences in neurodevelopment in a largely healthy cohort of children
**Methodological**	Issues pertaining to the use of an assessment tool in a multi-cultural sample should be addressed
	Issues pertaining to the acquisition of language skills in children with different native languages should be addressed
	Outcomes should be assessed as objectively as possibly, without rater, reporter and cultural biases
**Implementation and logistic**	Training of field workers in the administration of the selected tool should be easy and specialist training in pediatrics, psychology or neuroscience should not be a pre-requisite
	The assessments should be relatively inexpensive to administer
	The assessments should be rapid to administer to prevent over burdening field workers and spurious low scores due to child fatigue

### Selection of a Neurodevelopment Tool for the INTERGROWTH-21^st^ Project

The Infant Development Group selected a measure of neurodevelopment at 24 months using two sequential approaches, and the results of approach 1 informed the design and results of approach 2. The methods and results of each approach are described in the sections that follow.

#### Approach 1: Review of existing psychometric tools for assessing neurodevelopment at 24 months

The Infant Development Group compiled a list of the most well-established psychometric tools to measure neurodevelopment outcomes, including (but not limited to) cognition, language skills, motor skills and behavior, at 24 months in an international sample. This age was selected as it was found to be the earliest at which: (i) neurodevelopment is not confounded by transient neurological syndromes of prematurity and (ii) conventionally used developmental instruments, such as the Bayley Scales of Infant Development, have been found to possess an acceptable level of medium and long term predictive validity [18]. The list was compiled in two phases.

Phase 1 involved a search of online databases (PubMed, PsycINFO and Embase) using search terms for cognition, language, motor skills and behavior. These are listed in Table S1 in File S1.

During Phase 2, the Infant Development Group contacted a number of child development experts and authors of child psychometric measures from Bangladesh, Brazil, India, Italy, Kenya, the Netherlands, UK and USA. The experts were asked to list the available measures they were familiar with to assess cognition, language, motor skills and behavior at 24 months.

Phases 1 and 2 (Figure S1 in File S1) resulted in a list of 47 tools (Table S2 in File S1). Their purpose, the age ranges at which they may be used, the domains assessed and the languages the tools are currently available in are summarized in Table S2 in File S1. The Infant Development Group critically analyzed these tools against the criteria listed in Table 3. Thirteen fulfilled some of the Project's criteria. Information on the characteristics, purpose, and strengths and weaknesses (in to the context of the INTERGROWTH-21^st^ Project) of each tool is presented in Table S3 in File S1.

The 13 tools were critically analyzed against the Project's criteria by a scientific advisory panel of independent international experts in the fields of neurodevelopment, pediatric neurology, child psychiatry, child psychology, vision, neuroscience, ophthalmology, sleep research, perinatal medicine and epidemiology at a meeting organized by the Infant Development Group.

**Results of Approach 1:** The findings of Phases 1 and 2, as reviewed by the scientific advisory panel, resulted in four key conclusions. First, the panel acknowledged that no one tool fulfilled all the Project's criteria. Second, the panel found 5 of the 13 tools to individually fulfill most of the Project's criteria and recommended the use of these tools in their most sensitive and reliable domains of neurodevelopment (Table 5). These were the Bayley Scales of Infant Development – Third Edition (BSID) [19], [20], the Rapid Neurodevelopment Assessment (RNDA) [21], the Malawi Development Assessment Tool (MDAT) [22], the Griffiths Mental Development Scale [23], [24] and the Child Behaviour Checklist (CBCL) [25], [26]. Third, the panel recommended that while it is almost impossible for any psychometric instrument measuring ECD to be free from cultural biases, it is possible to undertake a decentering approach by selecting items that are suitable for middle- and upper-class families in high-, middle- and low-income contexts [27]. In this approach, no single cultural group provides the initial items sets. Instead the test development process sets out to select items that are equally familiar to the different target groups and excludes any materials or content that seems unfamiliar to any of the target groups. The rationale for this approach was that in the current global situation, children from most middle- and upper-class families across settings are exposed to a set of similar resources (such as cups, spoons, dolls, crayons, shoes, stacking blocks) and therefore it is possible to pool this common set of exposures to select a set of items that most middle- and upper-class children across high-, middle- and low-income settings can perform. Finally, the panel suggested a package-based approach combining the use of psychometric tools with neurophysiological measures. The panel recommended that the methodology, scope and sensitivity of such a package must be tailored to the needs of the INTERGROWTH-21^st^ Project.

**Table 5:** Neurodevelopment tools selected following review of candidate tools by scientific advisory panel.

Tool	Positive Features	Negative Features
**BSID-III**	• Considered to be the gold standard for psychometric evaluation of children <3 years of age	• Long administration time (60-90 minutes)
		• Specialist training required
	• Sensitive diagnostic instrument to detect subtle differences in neurodevelopment in a normal, healthy cohort	• Expensive
		• Concern about the cultural bias of items and norms
	• Good predictive and discriminant validity	
**24 month version of the RNDA**	• Quick and easy to administer	• Information on sensitivity, specificity and positive predictive value at 24 months not available
	• Yield continuous scores	
	• Used in LMIC samples	• Not validated in HIC settings
	• Administration does not require specialist training	• Not all items are free from cultural biases
	• Characterizes neurodevelopmental outcomes across a spectrum ranging from mild to severe delay	• Very few items in each domain
		• No evidence to indicate the measure would be suitable for use within an optimally healthy population
**MDAT**	• Quick and easy to administer	• Not validated in HIC settings
	• Yields continuous scores	• Not all items are free from cultural biases
	• Used in LMIC samples	• No evidence to indicate the measure would be suitable for use within an optimally healthy population
	• Administration does not require specialist training	
	• Strong items in the language domain	
**Griffiths Scales**	• Sensitive diagnostic instrument to detect subtle differences in neurodevelopment in a normal, healthy cohort	• Long administration time (60–90 minutes)
	• Validated in a number of international settings	• Specialist training required
	• Strong items in the gross motor domain	• Expensive
		• Not all items are free from cultural biases
**Pre-school version of the CBCL**	• Sensitive diagnostic tool to detect behavioral, emotional and attention problems	• Subject to reporter/recall bias
	• Validated in international settings and available in a large number of languages	• Does not assess cognitive, motor and language skills
	• Has a sleep and stress problems scale	• Some items may not be free from cultural biases
	• Short administration time (approximately 15 minutes)	

BSID  =  Bayley Scales of Infant Development III edition, RNDA  =  Rapid Neurodevelopment Assessment, MDAT  =  Malawi Development Assessment Tool, Griffiths Scales  =  The Griffiths Mental Development Scale, CBCL  =  Child Behavior Checklist

#### Approach 2: A multi-dimensional package for the assessment of neurodevelopment at 24 months

Based on the recommendations of the scientific advisory panel, the Infant Development Group undertook a structured, package-based approach to construct a neurodevelopment assessment protocol customized to the needs of the Project and the characteristics of the study population. The design of the Package was also guided by its potential for use as a population-based screening instrument for ECD in the context of LMICs and HICs. The process leading to the development of the Package was undertaken in five stages: (1) the neurodevelopment outcomes we wished to assess were grouped into similar domains; (2) a set of domain-specific criteria to be fulfilled by each neurodevelopment measure were developed; (3) the candidate measures that fulfilled the criteria for each domain were listed, based on the results of the systematic review, and discussions with experts and the scientific advisory panel; (4) the list of domain-specific, candidate measures was critically analyzed against the criteria for each domain and the most appropriate measure for each domain was selected by the scientific advisory panel and the Infant Development Group; and (5) the various measures were incorporated into a singular assessment package with domain-specific protocols.

**Results of Approach 2:** 1) Categorization of desired neurodevelopment outcomes into domains

Ten neurodevelopment outcomes were listed as priorities for the Project: cognition, language, gross motor skills, fine motor skills, behavior, attention, emotional reactivity, overall visual function, cortical auditory processing, and sleep. The rationale for the selection of these outcomes was each outcome has been previously found to have long-term predictive ability [7], [8], [28]–[30] and well-established psychometric, neurophysiological and/or clinical tests are available to measure them. The 10 outcomes were grouped into four domains based upon the balance between the technical, conceptual and logistical feasibility of assessing each domain on an international sample in accordance with the criteria and requirements of the INTERGROWTH-21^st^ Project. The domains are:

1. Vision: Visual acuity and contrast sensitivity
2. Neuropsychological function: Cognition, language, gross motor skills, fine motor skills, behavior, attention, emotional reactivity
3. Auditory function
4. Sleep

Language was included in domain 2 (neuropsychological function) rather than domain 3 (auditory function) in keeping with the contextual framework of the candidate measures. Furthermore, the expert panel felt it would be logistically more feasible to administer items on receptive and expressive language together with the cognitive, motor and behavior items and that this practice would contribute to a greater fluidity and continuity of the assessment for both the child and the assessor.

2) Domain-specific test selection criteria

A set of criteria to be fulfilled by a measure (psychometric, neurophysiological, clinical or a combination of these) for each domain was compiled by the Infant Development Group and approved by the scientific advisory panel. The test selection criteria for each domain are conceptually similar to the overall Project criteria depicted in Table 3. These are listed in detail in Tables S5–S8 in File S1.

3) Candidate measures for each domain

3.1) Vision

Conventional measures of vision in adults, such as the Snellen chart, require levels of comprehension, concentration and verbal skills that a young child may not possess. Therefore, in addition to the criteria listed in Table S4 in File S1, attention was paid to the candidate measures below being suitable for young children and free from biases introduced due to potential cognitive and language difficulties. These candidate measures, grouped as tests of visual acuity and contrast sensitivity, are listed below:

- Measures of Visual Acuity

Tests of visual acuity measure the child's ability to discern objects at a given distance according to a fixed standard. These include:

-
  - Tests using subjective/behavioral methods based on the forced choice preferential looking (FPL) method, conceived by David Teller [31]. In this method, the observer presents the child with a display, half of which is plain while the other half contains a pattern. The child tends to look at the pattern if he/she can resolve it. This technique becomes a “forced choice” when the observer has to decide, based on the child's head and eye movements, where the stimulus is located [32]. The threshold is defined as when the child makes a “clear” look. These methods consist of measures of resolution acuity (Teller acuity test [31], Keeler acuity test [33], Cardiff test [34], and grating and operant preferential looking techniques [35]) and measures of recognition acuity (Kay pictures, LEA symbols, Cambridge crowding cards, Landolt C-s and crowded Landolt C-s) [36], [37] .
  - Experiments using objective methods involving the measurement of pattern steady-state visual evoked potentials (VEP) and variations of the same (for example, sweep VEP) using electroencephalographic techniques [36].
- Measures of Contrast Sensitivity

Tests of contrast sensitivity assess the minimum contrast necessary for a subject to detect sine wave gratings of different spatial frequencies [35]. The measurement of contrast sensitivity is thought to reflect the integrity of the entire visual pathway, and is considered the “single most complete measure of human spatial vision” [37]. The measures include:

  - Tests using subjective/behavioral methods (Cardiff tests, Alley-running 2AFT staircase, Vistech charts and Pelli-Robinson Charts) [37].
  - Tests using objective methods, namely those measuring VEPs [36].

3.2) Neuropsychological function:

The findings of approach 1 were applied to the selection of candidate tools for the assessment of cognition, language, gross motor skills, fine motor skills, behavior, attention and emotional reactivity. Approach 1 has been described in detail above and the results of this process are enumerated in Tables S3 and S4 in File S1, and Table 5. Of the 47 measures initially identified, 13 were found to fulfill some of the Project's criteria, the top 5 of which were selected as candidate measures for use in the INTERGROWTH-21^st^ Project. These were the BSID [19], [20], the RNDA [21], the MDAT [22], the Griffiths scale [23], [24] and the CBCL [25] and were selected on the basis of their functionality to sensitively assess cognition, language, gross motor skills, fine motor skills and behavior at 24 months of age.

3.3) Auditory function:

There were five candidate tests for the assessment of auditory function as per the domain-specific criteria listed in Table S6 in File S1. These were audiometry, acoustic reflex threshold measurement, otoacoustic emission measurement, communication inventories and the measurement of evoked response potentials (ERPs) to auditory stimuli [38].

3.4) Sleep:

The candidate tools for sleep were classified by recording technique, as follows:

- Caregiver reports: These include sleep diaries and questionnaires. In the former, the caregiver maintains a diary recording of the child's activities, sleep and wake times and napping [39]. Sleep questionnaires assess the quality and frequency of the child's sleep [39], [40].
- Neurophysiological techniques: These include polysomnography (PSG) and actigraphy. PSG is the comprehensive measurement of the various biophysical changes that occur during sleep and involves the simultaneous recording of brain and heart signals, eye movements and muscle activity from multiple channels placed on the participant's head, face, limbs and chest [41]. The test is conventionally conducted in a sleep laboratory. Actigraphs are watch-like devices placed on the wrist or ankle to record movement [42]. The data are analyzed for periods of activity and inactivity to estimate sleep patterns and circadian rhythms [43].

4) Selection of test for each domain

4.1) Vision

The candidate tools for visual acuity and contrast sensitivity were assessed according to the specific criteria listed in4 in File S1. FPL techniques, particularly Cardiff Tests, Teller cards and Keeler cards, met the Project's criteria better than objective (neurophysiological) techniques (Table 6). A further analysis of the Cardiff, Teller and Keeler tests, presented in Table 7, showed the Cardiff tests to be the most appropriate for the purposes of the INTERGROWTH-21^st^ Project because (1) they measure both visual acuity and contrast sensitivity and (2) the images are more attention-grabbing and engaging for toddlers than the black and white pictures of the Teller/Keeler cards [33], [34], [44]. The Cardiff acuity and contrast sensitivity tests [34], [45] were, therefore, selected as the vision assessment of choice for the neurodevelopment package of the INTERGROWTH-21^st^ Project.

**Table 6:** Analysis of candidate vision tests against selection criteria.

Selection Criteria	Tests of vision	
**Essential Criteria:**	**Forced preferential looking techniques**	**Neurophysiological techniques**
1. The test must be suitable to assess vision in 2 year olds.	+	+
2. The test must assess the entire visual pathway and not merely specific components of the ophthalmic apparatus.	+	+
3. The test must be sensitive enough to detect subtle differences in vision in a healthy cohort of children.	+	+
4. The test must yield an objective score of vision.	+	+
5. The test must possess established validity	+	+
6. The measurement of vision must not be affected by disturbances in language development, cognition, and/or hearing.	+	+
7. The test must be suitable for use in LMICs and in low-resource settings, and should not contain items that are culture or language specific.	+	+
8. The duration of assessment for each individual child must not exceed 10 minutes.	+	-
9. It must be easy to train local field workers to administer the test and no specialist training in ophthalmology, pediatrics or related disciplines must be necessary.	+	-
**Desirable Criteria:**		
1. The test should require minimal infrastructure and any equipment to administer the test must not be expensive.	+	-
2. The test should assess more than one aspect of vision.	+	+

**Table 7:** Analysis of candidate FPL techniques to assess vision in young children.

Name of Test	Supplier	Visual faculties assessed	Apparatus	Method of administration	Established validity	Administration time	Ease of use	Price*
Cardiff acuity cards	Fogarty Associates	Visual acuity & contrast sensitivity	3 sets of 11 grey cards with six different pictures familiar to toddlers positioned at the top or bottom of the card. With the acuity cards, the pictures get progressively smaller in each set. In the contrast sensitivity cards, the pictures get progressively lighter in color in each set.	The child is seated on the parent's lap, 50 cm from the assessor. Two cards in each set are presented one after another and the assessor judges the position of the image based on the child's eye movements. The assessor checks to confirm this is correct – if so, he/she proceeds to the next level until there is a lack of correspondence between the child's eye movements and the position of the image [34], [44]	Yes	2.5 mins per eye	High	$850
Teller acuity cards	Vistech Consultants Inc., Dayton, Ohio	Visual acuity	17 25×56 cm cards, of which 15 cards contain a 12.5×12.5 cm patch of black-and-white, vertical square-wave grating matched to the surrounding grey to within 1% in space-average luminance [33].	Cards are presented to the child seated on the mother's/teacher's lap from a distance determined according to the child's age. The observer views the child's behavior through a central peephole to determine whether the child shows a preference to one side of the card [33].	Yes	5–10 minutes per eye	Medium	Unknown
Keeler acuity cards	Keeler Ltd, Windsor, Berkshire	Visual acuity	10 26.5×57.5 cm cards. Each card contains two 10.3 cm circular apertures, with centers located 15.5 cm to the left and the right of the central peephole. One aperture contains a grating while the other aperture is filled to be uniformly grey. Both apertures have a white border [33].	Same as above	Yes	Unknown	Medium	$2,224.00

*All prices mentioned are in USD and based on quotations obtained between April and June 2012.

4.2) Neuropsychological function

The analysis of the 5 candidate measures; namely the BSID [19], the RNDA [21], the MDAT [22], the Griffiths Scale [23], [24] and the CBCL [25]; against the domain specific criteria for the INTERGROWTH-21^st^ Project (Table S5 in File S1) is presented in Table 8. As none of the measures were found to meet all the Project's criteria, the scientific advisory panel and the Infant Development Group concluded that the Project should use a combination of the candidate tools, by integrating the strengths of some measures to complement the weaknesses of others. A 53-item tool, the INTERGROWTH-21^st^ Neurodevelopment Assessment (INTER-NDA; Table 9), was designed by including 30 cognitive, language and motor items; 6 behavioral items and 17 items measuring attention and emotional reactivity. These items were selected by international experts in child development who had extensive experience with the candidate measures and with the development of psychometric tools. The rationale for the selection of each individual item was based on the experts' agreement on (i) its suitability for the 22–26 month age group; (ii) its appropriateness for use in international populations using the decentering approach described earlier; (iii) its ability to be administered reliably and (iv) its ability to be adapted across cultural contexts. The administration and scoring of the items was adapted for the purpose of the Project and care was taken to ensure conceptual equivalence was maintained between the items of the INTER-NDA and those of the 5 candidate measures of neuropsychological function. A 5-point Likert scale was selected to objectively record the child's performance on each item with a view to characteristic levels of achievement for each item across a spectrum (rather than a binary pass/fail outcome measure). As raw scores for the candidate measures were not freely available it was not possible to select items following a statistical analysis of item weights. The assessment of behavior encompasses the assessor's observation of child's overall behavior during the assessment, and does not include reports on specific constructs of behavior such as temperament and empathy. The operation manual and training materials of the INTER-NDA are freely available at www.intergrowth21.org.uk/protocol.aspx?lang=1.

**Table 8:** Analysis of candidate cognitive, language, motor, behavior and attention tests against selection criteria.

Selection Criteria	Neurodevelopment Assessments/Questionnaires				
**Essential Criteria:**	**BSID**	**RNDA**	**MDAT**	**Griffiths**	**CBCL**
1. The test must be suitable to assess neurodevelopment in 2 year olds.	+	+	+	+	+
2. The test must assess the following aspects of neurodevelopment:					
(a) cognition	+	+	+	+	-
(b) language	+	+	+	+	-
(c) motor skills	+	+	+	+	-
(d) behavior	+	+	+	+	+
(e) attention	-	-	-	-	+
(f) emotional reactivity.	-	-	-	-	+
3. The test must be sensitive enough to detect subtle differences in neurodevelopment in a healthy cohort of children.	+	-	-	-	+
4. The test must characterize outcomes across a spectrum.	+	+	+	+	+
5. The test must possess established reliability and validity in international settings.	+	+	+	+	+
6. The test must be free from culture-specific items and suitable for use in both HICs and LMICs.	-	+	-	-	-
7. The test must be based upon objective reporting and not subjective judgment of the child's performance.	+/-	-	+/-	+/-	-
8. The duration of assessment for each individual child must not exceed 30 minutes.	-	+	-	-	-
**Desirable Criteria:**					
1. The test should employ a combination of methodologies including but not limited to direct tests, observation and caregiver reports.	+	+	+	-	-
2. The test should require minimal infrastructure and any equipment to administer the test must not be expensive.	-	+	+	-	+

**Table 9:** Constituent items and scoring sheet of the INTERGROWTH-21^st^ Neurodevelopment Assessment.

The INTERGROWTH-21^st^ Neurodevelopment Assessment (INTER-NDA)							
No.	Item	Observed performance					
1	**Builds a tower of 5 cubes (trials = 3, demonstration = 3)**	5 cubes	3–4 cubes		2 cubes		No attempt
2	**Names 4 colors when asked to do so (trials = 0)**	Names 4 colors	Names 3 colors		Names 1 or 2 colors		Does not name any color
3	**Matches 3 cubes of same colors when requested to do so (trials = 1, demonstration = 1 of one color)**	Matches 3 colors	Matches 2 colors		Matches 1 color		Does not match any color
4	**Hands the examiner one cube when asked to do so (Examiner says “Please give me one cube” & keeps palm open for 5 seconds after child has handed over 1 cube; trials = 1)**	Hands only one block within 5 seconds	Hands only one block in more than 5 seconds		Hands two or more blocks		Does not hand any block/No attempt
5	**Puts the spoon in the cup when asked to do so (trials = 5)**	Puts the spoon in cup in ≤3 trials	Puts the spoon in cup in ≤3 trials in 4–5 trials		Takes the spoon or the cup but does not complete action		No attempt
6	**Matches shapes on board (trials = 5, demonstration = 1 if 1^st^ trial unsuccessful)**	All shapes in ≤3 trials	All shapes with repeated demonstration i.e. 4–5 trials		One or two shapes 4–5 trials		No attempt
7	**Matches shapes on rotated board (trials = 5, demonstration = 1 if 1^st^ trial unsuccessful)**	All shapes in ≤3 trials	All shapes with repeated demonstration i.e. 4–5 trials		One or two shapes 4–5 trials		No attempt
8	**Points correctly when asked “Where is the door/entrance to the room?” (trials = 5)**	Identifies door correctly in ≤3 trials	Identifies door correctly in 4–5 trials		Attempts, but does not identify door		No attempt
9	**Puts a raisin precisely inside a small opening in a bottle (trials = 1, demonstration = 1; test both hands)**	Precise release of raisin into bottle with each hand	Clumsy release, raisin falls out of bottle with one or more hand		Attempts but unsuccessful release with one or more hand		No attempt
10	**Drinks water from cup/sippy cup when placed in front of child (trials = 1, maternal report if sippy cup unavailable)**	Drinks in a well-coordinated manner without spilling	Drinks clumsily & spills		Attempts but unsuccessful		No attempt
11	**Looks towards an object located across the room when pointed at by the examiner (trials = 5)**	Looks or points at object in ≤3 trials	Looks or points at object in 4–5 trials		Looks at the wrong object, or attempts but cannot identify object		No attempt
12	**Pretends to drink from the cup when a cup is placed in front of him/her (trials = 1, demonstration = 1)**	Spontaneous	After 1 demonstration		Partial attempt after 1 demonstration		No attempt
13	**Able to make a cup of tea with the toy tea set when requested by examiner (Examiner says “Can you make a cup of tea?”) (trials = 2, demonstration = 1 if 1^st^ trial unsuccessful)**	Spontaneous	After 1 demonstration		Partial attempt		No attempt
14	**Feeds doll when requested to (Examiner says “Can you give the dolly some tea?”) (trials = 2, demonstration = 1 if 1^st^ trial unsuccessful)**	Spontaneous	After 1 demonstration		Partial attempt after 1 demonstration		No attempt
15	**Imitates straight scribble (trials = 5, demonstration = 5)**	≤3 trials	4–5 trials; with difficulty		Attempts (hold crayon)		Cannot hold crayon
16	**Identifies glitter bracelet under correct washcloth (trials = 5, test both sides)**	Finds bracelet correctly in ≤2 trails on both sides	Find bracelet correctly in 3 trials or on one side only		Find bracelet correctly in 4–5 trials or on one side only		Does not find bracelet or no attempt
17	**Correctly identifies object groups using plurals**	Uses 5 plurals	Uses 3–4 plurals		Identifies 1–2 plurals		Does not use any plurals
18	**Asks for toilet by gesture or verbally ** ***(maternal report)***	Always	Occasionally		Partial (only for bowel movement)		Never
19	**Runs ** ***(maternal report)***	Runs steadily	Attempts		Walks only		Walks with support
20	**Throws a ball very near (trials = 1, demonstration = 1, test both sides)**	Good release	Unsteady release		Attempts		No attempt
21	**Kicks ball ** ***(maternal report)***	Kicks ball with flexed	Attempts		Walks only		No attempt
22	**Climbs upstairs holding rail, 2 feet/stair or in adult fashion ** ***(maternal report)***	Climbs stairs alone holding rail	Unsteady		Needs help		No attempt
23	**Uses 2**–**4 syllable babble such as dada, mama but not specifically to anything or any person**	Spontaneously	Mimics		1 syllable babble e.g. ba, ma, da		None
24	**Use two words together**	Yes, appropriate use	Yes, in appropriate use		One word		No attempt
25	**Indicates by gesture to say no ** ***(maternal report if not observed during assessment)***	Indicates in ≤3 trials	Indicates in 4–5 trials		Attempts, but incomplete indication		No attempt
26	**Use of a pronoun e.g. me, my, she, he, it, I**	Correct use	Incorrect use		No use		No use
27	**How many words does the child use during the assessment other than mama/dada**	≥8 words	6–7 words		4–5 words		≤3 words
28	**How many sentences of 3 words or more does the child use during the assessment?**	≥2	1		Only two – one word utterances		None
29	**In how many instances does the child follow on a topic of conversation providing new information?**	At least one, using ≥2 words, proving correct information	Uses single words, provides correct information		Uses any number of words, provides incorrect information		Does not follow up on conversations
30	**Combines word and gesture when asked (trials = 1, do not demonstrate)**	Yes, appropriate use & completed gesture	Yes, inappropriate use but complete gesture		Incomplete gesture and inappropriate use		None
**What is the child**'**s native (first) language?**							
**What is the language in which the assessment is being conducted?**							
**Does the child speak/understand any languages other than his/her native (first) language?**							
**How often were the following behaviors in the child during the assessment?**							
31	Positive Affect	Never or rarely		Some of the time		Most of the time	
32	Exploration	Never or rarely		Some of the time		Most of the time	
33	Ease of engagement	Never or rarely		Some of the time		Most of the time	
34	Cooperativeness	Never or rarely		Some of the time		Most of the time	
35	Adaptability to change	Never or rarely		Some of the time		Most of the time	
36	Distractibility	Never or rarely		Some of the time		Most of the time	
37	Negative affect	Never or rarely		Some of the time		Most of the time	
**Caregiver Reported Child Behaviour Questionnaire**							
Instructions to caregiver: Please fill in this form to reflect your view of your child's behavior, even if others do not agree							
38	Likes playing with other children	Not true		Sometimes true		Often true	
39	Can't concentrate, can't pay attention for long	Not true		Sometimes true		Often true	
40	Can't sit still, restless or hyperactive	Not true		Sometimes true		Often true	
41	Disturbed by any change in routine	Not true		Sometimes true		Often true	
42	Nervous movements or twitching	Not true		Sometimes true		Often true	
43	Shows panic for no good reason	Not true		Sometimes true		Often true	
44	Poorly coordinated or clumsy	Not true		Sometimes true		Often true	
45	Quickly shifts from one activity to another	Not true		Sometimes true		Often true	
46	Rapid shifts between sadness and excitement	Not true		Sometimes true		Often true	
47	Sudden changes in mood or feelings	Not true		Sometimes true		Often true	
48	Sulks a lot	Not true		Sometimes true		Often true	
49	Upset by new people or situations	Not true		Sometimes true		Often true	
50	Wanders away	Not true		Sometimes true		Often true	
51	Whining	Not true		Sometimes true		Often true	
52	Worries	Not true		Sometimes true		Often true	
53	Responds well to affection	Not true		Sometimes true		Often true	
**END**							

4.3) Auditory function

The candidate tools for auditory function were analyzed according to the domain specific criteria (Table 10, Table S6 in File S1). Audiometry and the measurement of auditory ERPs were the only tests found to: (i) be sensitive enough to detect subtle differences in auditory function in a cohort of healthy children and (ii) assess the entire auditory pathway [46]. Audiometry was, however, found to be difficult to perform reliably in children less than three years of age [46], [47]. Although the measurement of acoustic reflex thresholds and otoacoustic emissions detect the most common causes of hearing impairment in children they solely assess middle ear function, and not the entire auditory pathway [48]. In addition, these tests require a probe to be placed in the child's ear for up to 3 minutes, which would pose considerable difficulties with compliance in the field [48].

**Table 10:** Analysis of candidate auditory function tests against selection criteria.

Selection Criteria	Measures of auditory function				
**Essential Criteria:**	**Audiometry**	**Acoustic Reflex Thresholds**	**Otoacoustic Emissions**	**Communication Inventories**	**Auditory ERPs**
1. The test must be suitable for assessing auditory function in children at 2 years of age.	-	+	+	+	+
2. The test must assess the entire auditory pathway and not merely specific components of the auditory apparatus.	+	-	-	-	+
3. The test must be sensitive enough to detect subtle differences in auditory function in a healthy cohort of children.	+	+	+	-	+
4. The technique must possess established validity and be a well-established measure of auditory function in children.	+	+	+	+	+
5. The measurement of auditory function must not be affected by simultaneous disturbances in cognition, and/or vision.	+	+	+	-	+
6. The technique must be suitable for use in LMICs and in low-resource settings, and should not contain items that are culture or language specific.	+	+	+	-	+
7. The duration of assessment for each individual child should not exceed 20 minutes.	-	+	+	+	+
8. It must be easy to train local field workers to administer the test and no specialist training in ophthalmology, pediatrics or related disciplines should be necessary.	-	-	-	+	-
**Desirable Criteria:**					
1. Any equipment to administer the technique must not be expensive or difficult to use.	-	-	-	+	-
2. The technique should yield objective measures of amplitude and latency of responses.	+	+	-	-	+

The measurement of auditory ERPs using the novelty oddball ERP task [49] was the method of choice for the Project. This technique has been validated in young children and tests their ability to discriminate novel stimuli embedded in a train of frequent stimuli [49]. It involves the presentation of frequent, infrequent and novel auditory stimuli, and the measurement of the amplitude and latencies of distinct EEG waveforms to each stimulus. The waveforms of interest include the P1 (a positive peak around 100 ms after stimulus onset), the N2 (a negative peak around 200 ms) and the P3a (a positive peak around 250–350 ms).

However, measuring auditory ERPs involves recording EEG signals from the child's scalp. This is complicated because it requires specialist training, sophisticated equipment, long set-up times and possibly sedation of the child [46]. The Infant Development Group had specific concerns about the co-operation of children, the consent of mothers and the ease of implementation of this technique in the field, especially in low resource settings, with conventional EEG apparatus and methods. To meet these challenges, the Group designed a novel method of measuring auditory ERPs in children employing a ‘strap and click’ design using a wireless EEG cap and dry electrodes [50].

4.4) Sleep

The candidate tools to measure sleep in children were analyzed according to domain specific criteria (Table S7 in File S1, Table 11). Actigraphy was found to be the only method of assessment that was: (i) of sufficient sensitivity to measure subtle differences in sleep in a healthy cohort of children, (ii) objective, (iii) minimally disruptive to the daily routine of the mother and child, and (iv) able to record sleep patterns and daytime physical activity in a natural, home based setting [42], [43]. However, leading actigraphy and sleep experts on the scientific advisory panel felt that actigraphy alone may not adequately capture the child's sleeping pattern as the information collected is limited by the number of days that the child wears the actiwatch. It was therefore decided that the use of a well-established maternally reported sleep questionnaire, such as the Brief Infant Sleep Questionnaire [51], in conjunction with actigraphy may be a more reliable method of assessing sleep and daytime activity patterns in children than either alone.

**Table 11:** Analysis of candidate sleep measures against selection criteria.

Selection Criteria	Measures of sleep & circadian rhythm			
**Essential Criteria:**	**Caregiver reports**		**Neurophysiological methods**	
	**Sleep Diary**	**Sleep Questionnaire**	**Polysomno-graphy**	**Actigraphy**
1. The test must be suitable for assessing sleep in children at 2 years of age.	+	+	+	+
2. The test must be sensitive enough to detect subtle differences in sleep in a healthy cohort of children.	+/−	+/−	+	+
3. The test must possess established validity and be a well-established measure of sleep in children.	+	+	+	+
4. The test must be an objective measure of sleep in children.	-	-	+	+
5. The test must be as non-disruptive to daily life as possible for both the child and the caregivers, and should not require prolonged periods of recording in laboratories.	+	+	-	+
6. The test must measure sleep in a natural, home based setting.	+/−	+/−	-	+
7. The test must be suitable for use in LMICs as well as HICs.	+/−	+	-	+
8. t must be easy to train local field workers to administer the tool and no specialist training in pediatrics, child psychiatry, neuroscience or related disciplines must be necessary.	+	+	-	+
**Desirable Criteria:**				
1. Any equipment to administer the technique should not be expensive.	+	+	-	+/−
2. The technique should yield information on a range of sleep characteristics including but not limited to sleep efficiency, total duration of sleep and nighttime awakenings.	-	-	+	+
3. The technique should yield information about the extent of the child's motor activity during the day.	-	-	-	+

## Results and Discussion

### The INTERGROWTH-21^st^ Neurodevelopment Package

The INTERGROWTH-21^st^ Neurodevelopment Package describes a multi-dimensional, mixed-methodology approach to assessing neurodevelopment in preschoolers. The Package is the result of the combined efforts of the Infant Development Group and the scientific advisory panel to develop a holistic, scalable instrument to measure ECD that is easy to implement across different contexts and settings for population-based research and screening purposes. The Package, in its entirety, meets all the Project's requirements listed in Table 3, while each individual component of the Package fulfills domain specific criteria listed in Tables 6, 8, 10 and 11.

### Characteristics of the Package

**Figure 3:**
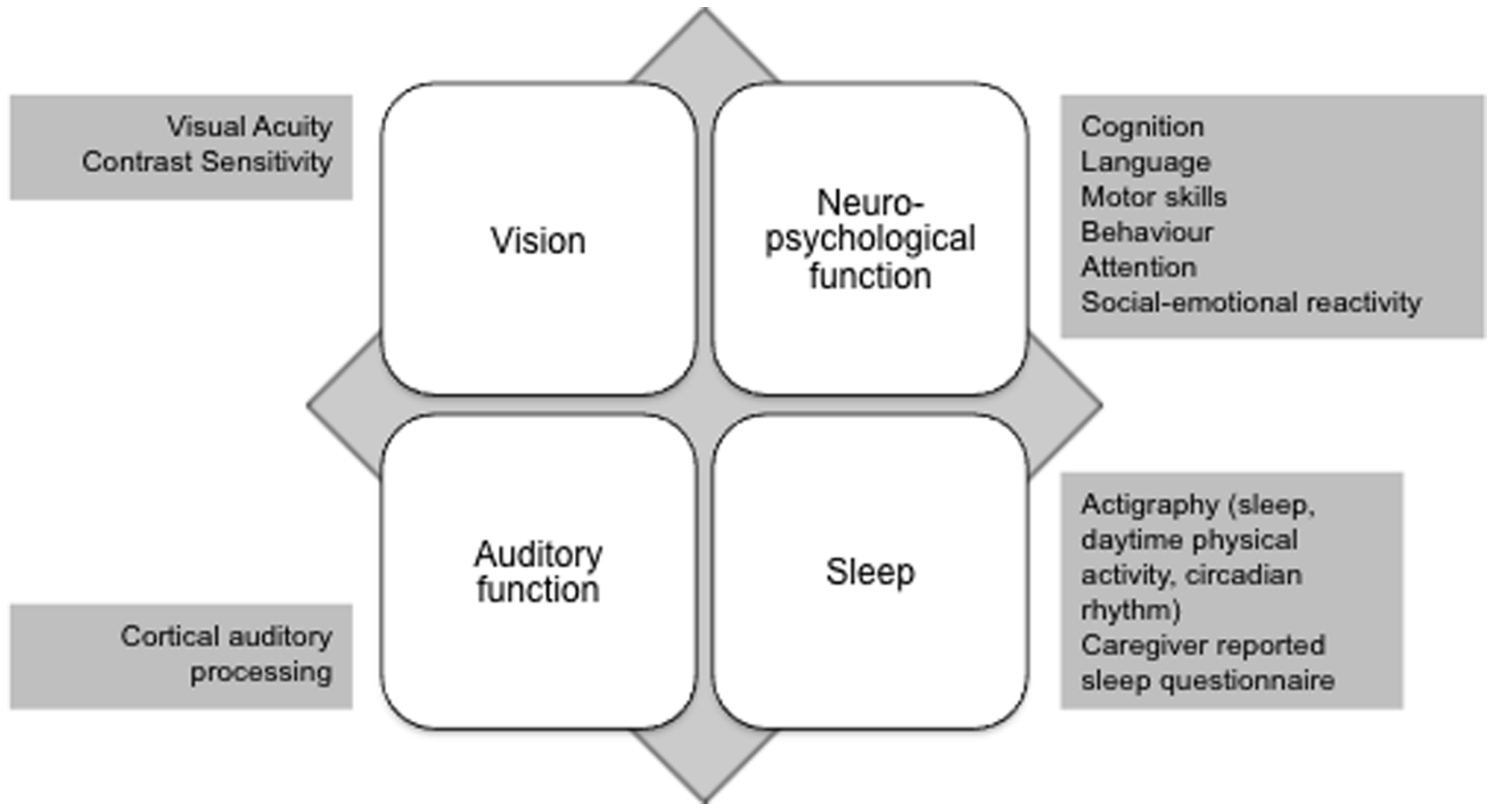
Components of the INTERGROWTH-21^st^ Neurodevelopment Package.

The package consists of four constituent assessments (Fig. 3). These include a test of vision; a measure of cognition, language, motor skills, behavior, attention and emotional reactivity; a test of cortical auditory processing and a measure of sleep patterns and daytime physical activity. The package takes between 35 and 45 minutes to administer and consists of a combination of psychological, neurophysiological and clinical tests, and caregiver reports.

### Key Features

The important features of the Package are that it:

1. Is a multi-dimensional assessment of neurodevelopment in preschoolers using a mixed methodology approach
2. Was developed for, and is currently in use in, middle- and upper-class children across low-, middle- and high- income settings
3. Can be delivered in the field by non-specialist staff with minimal training
4. Yields objective, rather than subjective, information about a child's performance
5. Incorporates the assessment of entire vision and auditory pathways rather than individual anatomical/physiological components of these pathways.

### Implementation of the INTERGROWTH-21^st^ Neurodevelopment Package

#### Automation

A number of components of the Package were automated to facilitate rapid administration, real-time data upload and integrated quality checks in the data collection system. A tablet-based data collection application, the NeuroApp, was designed for this purpose. The NeuroApp is an intuitive user interface with integrated operation manuals. It consists of: (a) a biography page, (b) a neurodevelopment home page providing access to the tests for the four constituent domains, and (c) vision, INTER-NDA, cortical auditory processing, and sleep pages on which the child's performance on each individual test are recorded. Integrated quality checks ensure that no more than one option is selected for each item and any items with missing data are flagged before the data upload stage.

To ensure that non-specialist research staff can carry out the cortical auditory processing experiment, a ‘strap-and-click’ design was developed. This method involved hardware and software customization of the EEG system (Enobio, Neuroelectrics, Barcelona) to automate the presentation of the auditory stimuli and the recording, termination and upload of the corresponding electrophysiological data following a simple initial set-up.

The Package's tri-axial actigraph of choice, marketed as the Motionwatch 8 [52], is reliable under harsh climate conditions, waterproof, easy to set up and use, and has a relatively long battery life. It does not require calibration and data are easy to download onto a computer using the Motionware software. The data are uploaded onto a dedicated sleep section on the Project's centralized database using a standard file transfer protocol.

In order to ensure that non-specialist research staff involved in the study are capable of conducting psychological, neurophysiological and clinical tests to the same degree of proficiency as specialist research staff a comparison of the data quality obtained between the two will be carried out following the conclusion of data collection. Although there is considerable doubt among the scientific community about the validity of neurodevelopment assessments being carried out by non-specialist research staff with minimal training, the INTERGROWTH-21^st^ Project offers the opportunity to test the hypothesis that with targeted training, user-friendly apparatus and a high degree of automation (including integrated quality checks), non-specialist field workers may be able to achieve levels of proficiency at power with specialist research staff. We believe this would be an important step ahead in the field where the lack of trained personnel is often the rate-limiting step in the scalability of ECD screening packages, especially in LMICs.

#### Database organization

Neurodevelopment data from all study sites are stored securely in a dedicated neurodevelopment section on the Project's centralized, cloud-based database. Neuropsychological and vision data (in.txt format) are uploaded instantaneously from the NeuroApp following the completion of an assessment. EEG data (in.zip, info and.easy formats) are uploaded automatically from the EEG software. Actigraphy data (in.awd format) are uploaded through a standard file transfer protocol. The database for the Project was designed, and is maintained, by MedSciNet, (Stockholm, Sweden) and managed by local and coordinating unit data managers with varying levels of access and administrative rights. Information on the organization of the database, data access and security is available elsewhere [53].

#### Training

The Infant Development Group devised the training strategy for the Package by taking into consideration the requirements of each site. The training session, which lasts 3½ days, is conducted at each site by the coordinator of the Infant Development Group. Day 1 consists of 6 sessions where trainees are introduced to, and trained in, the various theoretical and practical aspects of Package using a combination of direct instruction, small group interaction, role-play and problem-based learning. Day 2 consists of 9 sessions – 6 refresher sessions, a demonstration and 2 live sessions. Day 3 consists of 3–4 live sessions and a video based standardization session to assess inter-rater and test-re test reliability. The half-day consists of a further live session, written test and discussion about the standardization session. At the end of every live assessment, the trainees are encouraged to discuss the assessment with the trainer and give feedback. Specific attention is paid to problem areas and site-specific cultural issues at this point. The rationale for conducting on-site, rather than centralized, training sessions is to enable the trainer to help the teams with site-specific issues and to test the apparatus. It also provides an opportunity for the trainer to liaise with the IT and data experts at the sites and to troubleshoot initial problems.

#### Cultural customization

At each training session, the site team together with the coordinator of the Infant Development Group compiles a list of item-based culture-specific issues for the site and discusses solutions to address these problems. The issues largely concern the amendment of phrases during the administration of the INTER-NDA to more culturally-appropriate conceptual equivalents (for example, in item 30 on combining a word and gesture, it was found that certain phrases such as ‘show me Gota’ in Kenya, and ‘do Namaste’ in India, worked better than English versions such as ‘blow a kiss’ or ‘wave bye’). For maternally reported items on attention and emotional reactivity, local language versions (Brazilian Portuguese, Hindi, Italian, Kiswahili and Marathi) from validated CBCL translations were made available to the site staff.

### Reliability Analysis and Comparison with Other Neurodevelopment Scales

The inter-rater and test-re test reliability of the INTER-NDA was determined across 21 assessors in Brazil, India, Italy, Kenya and the UK. This was carried out using video recordings of INTER-NDA assessments on four children performed by an expert assessor (MF). All assessors were instructed to simultaneously score each child according to a standardized reliability protocol. Cohen's kappa coefficients of reliability were calculated. These were found to be substantial for both inter-rater reliability (k = 0.70, 95% CI: 0.47–0.88) and test-re test reliability (k = 0.79, 95%CI: 0.48–0.96).

A study to evaluate the agreement between the INTER-NDA and the BSID-III is currently underway at our Oxford site. The results of this analysis will be published separately.

### Progress to date

The INTERGROWTH-21^st^ Project teams in Brazil, Kenya, India, Italy and the UK were trained between November 2012 and September 2013. Implementation of the Package commenced in the UK in February 2013, followed by Kenya, India and Italy in April 2013 and Brazil in November 2013. To date, 829 children in these sites have been assessed.

The Package takes between 35 and 45 minutes to administer in the field. The duration of each component of the Package is as follows: vision, 5 minutes; INTER-NDA, 15–20 minutes; cortical auditory processing, 12 minutes, and sleep, 3–5 minutes. The mean administration times in each site, rounded to the nearest 5 minutes, are as follows, – Brazil: 40 minutes, India: 45 minutes, Italy: 45 minutes, Kenya: 40 minutes and the UK: 35 minutes.

The Package was found to be easy to administer in all sites. Technical difficulties including electricity failures during ERP recordings and Internet connectivity problems occurred to a small extent in all sites. The HIC sites witnessed greater difficulty in persuading children to keep the wireless EEG cap on for the entire duration of 10 minutes as compared to the LMIC sites. The return of the actiwatch to the study center by post proved to be difficult in the Indian, Italian and Kenyan sites and alternative methods of delivery, such as courier services, were therefore organized.

## Discussion

The INTERGROWTH-21^st^ Neurodevelopment Package describes, to the best of our knowledge, the first multi-dimensional instrument for early child development, for both research and screening purposes, that is designed to be carried out by non-specialist assessors and that has been developed for use in children from middle- and upper class families across low-, middle- and high-income settings. The Package assesses visual acuity and contrast sensitivity (with the Cardiff tests); cognition, language, motor skills, behavior, attention and emotional reactivity (the INTER-NDA); cortical auditory processing (measuring auditory ERPs to a novelty oddball paradigm) and sleep-wake patterns and daytime physical actigraphy (actigraphy and maternal reports). The Package is in use in the INTERGROWTH-21^st^ study sites in Asia, Africa, Europe and South America where more than 700 children have been assessed. The operation manuals and protocols are freely available at www.intergrowth21.org.uk/protocol.aspx?lang=1. This paper describes the conceptual and methodological challenges encountered during the selection of multi-dimensional ECD measures and outlines the methodological process of systematic evaluation and tool selection undertaken by the INTERGROWTH-21^st^ Project towards the development of the Package. The paper also presents an overview of, to our knowledge, the most well-established psychometric measures of ECD for preschoolers. We believe that both the description of the systematic approach leading to the development of the Package, and the findings of the overview of psychometric tools to assess ECD in preschoolers, may be of use to researchers, policy makers and organizations involved in ECD research and surveillance at local, national and global levels when selecting and/or designing multi-dimensional, context-dependent, population-based instruments for measuring ECD.

The Package has a number of strengths. First, is its ability to assess multiple dimensions of neurodevelopment in preschoolers without the need for specialist staff or infrastructure. Second, the package was designed to reduce cultural and language biases in its administration as much as possible, thus making it suitable for use among children from middle- and upper class families across low-, middle- and high-income settings. This was done by selecting a pool of items using a decentering approach which, to the best of the Group's knowledge, involved resources that were familiar to children from middle- and upper-class families across settings and were not affected by cultural and language biases. The Group also adopted a scoring system based on objective observations of the child's performance rather than subjective judgments and undertook a process of cultural customization during on-site training sessions. Third, non-specialist field workers can easily deliver the Package in 35–45 minutes, and integrated quality checks in tablet-based application ensure that data can be collected reliably across diverse settings and contexts. An added feature of the Package is that its constituent assessments measure the function of entire vision and auditory pathways and not merely specific components.

There are a number of methodological and conceptual limitations to consider. First, the selection of tools for the Package was based on an overview of existing well-established instruments to assess ECD in preschoolers. This process consisted of a systematic review of the literature and discussions with experts, and not on a meta-analysis of existing tools, which might have produced a more objective assessment. The results of this process resulted in a list of, to our knowledge, the most well-established psychometric tools to assess children at 2 years of age but we acknowledge that this list is not exhaustive. In addition, the results of the systematic review were not evaluated against the PRISMA or STROBE guidelines [54], [55] because our primary concern was not the quality of the individual studies but the suitability of the instrument for the Project. Second, it may appear that the INTER-NDA is based on a variety of items from a number of different tools. However, all items are conceptually equivalent to those in the candidate measures and were carefully selected by international experts for the purpose of the study and the tool's future potential as a population-based screening instrument of ECD. Third, the INTER-NDA is not yet available in all the languages of the participating study sites, although translations are being prepared in accordance with the WHO Mental Health Initiative translation guidelines [56]. The Package was designed specifically for the 22 to 26 month age group and should not be used, therefore, in other age groups. Fourth, the tool was designed for use among children from middle- and upper class families, and its usefulness needs to be assessed in children from poorer backgrounds. Lastly, the Package does not assess the nature and degree of the concerns of caregivers about their children in detail.

## Conclusions

To the best of our knowledge, the Package represents the first attempt at a concise, yet comprehensive, multi-dimensional instrument to measure early child development by non-specialist field workers across low- middle- and high-income settings. The comprehensiveness of the Package, its mixed methodology approach and ease of use make it a good candidate for scalability in the context of population-based research, as well as for screening purposes. This paper describes the current landscape of well-established psychometric tools to measure ECD in preschoolers and outlines the methodological approach of systematic evaluation and tool selection, which led to the development of the Package. We believe this approach may be of use to persons and organizations involved in large-scale efforts in ECD research and surveillance when selecting and/or developing measures of ECD.

## Supporting Information

File S1 **Combined supporting information file containing all supporting figures and tables.** Figure S1, Process and results of the systematic literature review of existing psychometric tools for assessment of neurodevelopment at 24 months. Table S1, List of search terms to identify the most well-established psychometric tools in a search of online databases (Approach 1, Phase 1). Table S2, Summary of well-established neuropsychological measures to assess neurodevelopment in children at 24 months. Table S3, Shortlisted neurodevelopmental assessment tools for use in children at 24 months. Table S4, Selection criteria for vision tests. Table S5, Selection criteria for neuropsychological tests (cognition, language, motor skills, behavior, attention and emotional reactivity). Table S6, Selection criteria for auditory function tests. Table S7, Selection criteria for sleep tests.
(DOCX)
