# Supplementary material for: The INTERGROWTH-21st Project Neurodevelopment Package: A Novel Method for the Multi-Dimensional Assessment of Neurodevelopment in Pre-School Age Children
Source: PLoS One. 2014 Nov 25;9(11):e113360. doi: 10.1371/journal.pone.0113360 (PMC4244160; doi:10.1371/journal.pone.0113360)
Supplement: File S1 — Combined supporting information file containing all supporting figures and tables. Figure S1, Process and results of the systematic literature review of existing psychometric tools for assessment of neurodevelopment at 24 months. Table S1, List of search terms to identify the most well-established psychometric tools in a search of online databases (Approach 1, Phase 1). Table S2, Summary of well-established neuropsychological measures to assess neurodevelopment in children at 24 months. Table S3, Shortlisted neurodevelopmental assessment tools for use in children at 24 months. Table S4, Selection criteria for vision tests. Table S5, Selection criteria for neuropsychological tests (cognition, language, motor skills, behavior, attention and emotional reactivity). Table S6, Selection criteria for auditory function tests. Table S7, Selection criteria for sleep tests. (DOCX) [file pone.0113360.s001.docx]

**Supporting Information**

Table S1 List of search terms to identify the most well-established psychometric tools in a search of online databases (Approach 1, Phase 1).

| **Neurodevelopment Domain** | **Search terms** |
| --- | --- |
| **Cognition** | ‘Cognitive development’ OR ‘neurodevelopment*’ OR ‘developmental disability’ OR ‘developmental disabilities’ OR ‘development* delay’ OR ‘learning disability’ OR ‘learning delay’ OR ‘neurodevelopment* impairment’ OR ‘developmental disorder’ OR ‘neurodevelopment* disorder’ OR ‘Bayley Scales of Infant Development’ OR ‘Bayley test*’ OR ‘assessment*’ OR ‘Screening’ OR ‘tool*’ OR ‘questionnaire*’ OR ‘report*’ OR ‘batter*’ OR ‘scale*’ OR ‘measure*’ |
| **Language** | ‘Language development’ OR ‘language*’ OR ‘expressive*’ OR ‘receptive*’ OR ‘language skill*’ OR ‘speech*’ OR ’speak*’ AND ‘development*’ OR ‘acquisition*’ OR ‘delay*’ OR ‘impairment*’ OR ‘disability’ OR ‘disabilities’ OR ‘disorder*’ AND ‘Bayley Scales of Infant Development’ OR ‘Bayley test*’ OR ‘assessment*’ OR ‘Screening’ OR ‘tool*’ OR ‘questionnaire*’ OR ‘report*’ OR ‘batter*’ OR ‘scale*’ OR ‘measure*’. |
| **Motor Skills** | ‘Motor development’ OR ‘motor milestones*’ OR ‘fine motor*’ OR ‘gross motor*’ OR ‘motor* skill*’ OR ‘ambulat*’ OR ‘activity’ OR ‘action’ OR ‘activities’ AND ‘development*’ OR ‘acquisition*’ OR ‘skill*’ OR ‘delay*’ OR ‘impairment*’ OR ‘disability’ OR ‘disabilities’ OR ‘disorder*’ AND ‘Bayley Scales of Infant Development’ OR ‘Bayley test*’ OR ‘Griffiths Scales’ OR ‘WHO milestones’ OR ‘assessment*’ OR ‘Screening’ OR ‘tool*’ OR ‘questionnaire*’ OR ‘report*’ OR ‘batter*’ OR ‘scale*’ OR ‘measure*’. |
| **Behavior** | Behav*’ OR ‘conduct*’ OR ‘response*’ OR ‘irritab*’ OR ‘performance*’ OR ‘react*’ AND ‘development*’ OR ‘problem*’ OR ‘impairment*’ OR ‘disturbance*’ OR ‘difficulty’ OR ‘difficulties’ OR ‘disorder*’ AND ‘Bayley Scales of Infant Development’ OR ‘Bayley test*’ OR ‘Griffiths Scales’ OR ‘Child Behavior Checklist*’ OR ‘Screening’ OR ‘tool*’ OR ‘questionnaire*’ OR ‘report*’ OR ‘batter*’ OR ‘scale*’ OR ‘measure*’ |

Figure S2 Process and results of the systematic literature review of existing psychometric tools for assessment of neurodevelopment at 24 months

Discussion

with

authors and experts

Literature review of all available tools to measure neurodevelopmental outcomes at 24 months

42 tools

47 tools

47 tools

Critical analysis against Project’s criteria

Shortlist of candidate 13 tools

Review of tools by scientific advisory panel

Selection of 5 potentially suitable tools

Concept of a package

Table S3 Summary of well-established neuropsychological measures to assess neurodevelopment in children at 24 months.

| **No.** | **Name** | **Age Range** | **Purpose** | **Domain Assessed** | | | | | | **Languages available** |
| --- | --- | --- | --- | --- | --- | --- | --- | --- | --- | --- |
|  |  |  |  | **Cognitive** | **Language** | **Math** | **Motor** | **Adaptive** | **Social Emotional** |  |
| 1 | Adaptive Behavior Assessment System | 0-17 years | D | X | X |  | X | X | X | English  Spanish |
| 2 | Ages & Stages Questionnaire | 4 months – 5 years | S | X | X |  | X | X | X | English, Spanish, French, Korean |
| 3 | Arizona Articulation Proficiency Scale | 1.5 year – 18 years | D |  | X |  |  |  |  | English |
| 4 | Battelle Developmental Inventory | 0-7 years | S & D | X | X |  | X | X | X | English  Spanish |
| 5 | Bayley Scales of Infant and Toddler Development | 1-3.5 years | D | X | X |  | X | X | X | English |
| 6 | Behavioral Assessment System for Children | 2 years – college | D |  |  |  |  |  | X | English  Spanish |
| 7 | Brief Infant Toddler Social and Emotional Assessment | 1-3 years | S |  |  |  |  |  | X | English  Spanish |
| 8 | Brigance Early Preschool Screen | 2-3 years | S | X | X |  | X | X | X | English  Spanish |
| 9 | Carey Temperament Scales | 1-12 years | S |  |  |  |  |  | X | English |
| 10 | Carolina Curriculum for Infants and Toddlers with Special Needs | 2-5 years | S | X | X |  | X | X | X | English |
| 11 | Child Behavior Check list | 0.5 – 18 years | S and D | X – Attention only |  |  |  |  | X | 94+ languages & dialects, including all major world languages |
| 12 | Communication & Symbolic Behavior Scale Developmental Profile | 6 months – 6 years | S |  | X |  |  |  | X | English |
| 13 | Creative Curriculum Developmental Curriculum for Infants, Toddlers and twos | 0-<2 years | S | X | X |  | X |  | X | English  Spanish |
| 14 | Developmental Assessment of Young Children | 0 – <6 years | D | X | X |  | X | X | X | English |
| 15 | Developmental Observation Checklist System | 0-6 years | S | X | X |  | X |  | X | English |
| 16 | Devereux Early Childhood Assessment Development Program | 2-5 years | S |  |  |  |  |  | X | English |
| 17 | Expressive One-Picture Vocabulary Test | 2-18 years | D |  | X |  |  |  |  | English |
| 18 | Eyberg Child Behavior Inventory | 2-6 years | S |  |  |  |  |  | X | English |
| 19 | Griffiths Mental Developmental Scales | 0-8 years | D | X | X |  | X |  | X | English |
| 21 | Hawaii Early Learning Profile | 0-3 years | S | X | X |  | X | X | X | English  Spanish |
| 22 | Individual Growth and Development Indicators for Infants and Toddlers | 0-3 years | S and D | X | X |  | X |  | X | Any |
| 23 | Infant Toddler Developmental Assessment | 0-3 years | D | X | X |  | X | X | X | English  Spanish |
| 24 | Infant Toddler Environment Rating Scale - Revised | 0-2 years 6 months | D | X | X |  | X | X | X | English, Spanish, German, Japanese |
| 25 | Infant Toddler Sensory Profile | 0-3 years | D |  |  |  |  |  |  | English  Spanish |
| 26 | Infant Toddler Social and Emotional Assessment | 1-3 years | D |  |  |  |  |  | X | English  Spanish |
| 27 | Infant Toddler Symptom Checklist | 7 months – 2.5 years | S | X | X |  | X | X | X | English |
| 28 | Khan-Lewis Phonological Analysis | 2-21 years | D |  | X |  |  |  |  | English |
| 29 | Kilifi Developmental Inventory | 6-35 months | S | ?X |  |  | X |  |  | Kiswahili |
| 30 | MacArthur-Bates Communicative Development Inventories | 8 months – 3 years | D |  | X |  |  |  |  | English |
| 31 | Merrill-Palmer Revised Scales of Development | 0-6 years 6 months | D | X | X |  | X | X | X | English  Spanish |
| 32 | Mullen Scales of Early Learning | 0-5 years 8 months | S | X | X |  | X |  |  | English |
| 33 | Malawi Developmental Assessment Tool | 0-6 years | S |  | X |  | X |  | X | Malawi |
| 34 | Ounce Scale | 0-3.5 years | S | X | X |  | X |  | X | English  Spanish |
| 35 | Parent’s Evaluation of Developmental Status | 0-8 years | S | X | X |  | X | X | X | English, Spanish, Vietnamese, Chinese |
| 36 | Pervasive Developmental Disorders Screening Test | 1-4 years | S |  | X |  |  |  | X | English  Spanish |
| 37 | Preschool Language Scale | 0-6 years | D |  | X |  |  |  |  | English  Spanish |
| 38 | Rapid Neurodevelopmental Assessment | 0 – 5 years | S | X | X |  | X | X | X | English  Bengali |
| 39 | Receptive Expressive Emergent Language Scale | 0-3 years | D |  | X |  |  |  |  | English |
| 40 | Scales of Independent Behavior | 3 months – 80 years | S & D |  | X |  | X | X | X | English |
| 41 | Stanford-Binet Intelligence Scales for Early Childhood | 2-7 years | D | X |  |  |  |  |  | English |
| 42 | Temperament and Atypical Behavior Scale | 1-5 years | S & D |  |  |  |  |  | X | English |
| 43 | Test of Early Language Development | 2-7 years | D |  | X |  |  |  |  | English |
| 44 | Toddler & infant motor evaluation | 0-3.5 years | D |  |  |  | X | X | X | English |
| 45 | Transdisciplinary Play Based Assessment | 0-6 years | S | X | X |  | X |  | X | English |
| 46 | Vineland Adaptive Behavior Scales | 0-90 years | D |  | X |  | X | X | X | English  Spanish |
| 47 | Woodstock Johnson III NU complete | 2-90 years | D | X | X | X |  |  |  | English  Spanish |

S = screening instrument, D = diagnostic instrument

Table S4 Shortlisted neurodevelopmental assessment tools for use in children at 24 months.

| Name of Scale | Construct assessed | Age Range | Adminis-tration time (minutes) | Type of Instrument | Key Advantages | Key Disadvantages |
| --- | --- | --- | --- | --- | --- | --- |
| Screening Instruments | | | | | | |
| Ages & Stages Questionnaire (ASQ)^[[1]](#footnote-1)^ | Infant/Child Development (communication, gross motor, fine motor, problem-solving, and personal-social) | <5 years | 10-15 | Parent/caregiver report, screening tool. | Easy to use in many settings, well standardized. | Cultural specific items, subject to reporter & recall bias. |
| The Brief Infant-Toddler Social & Emotional Assessment (Brief ITSEA)^[[2]](#footnote-2)^ | Internalizing, externalizing, dysregulation & competence | 1-3 years | 7-10 | Parent/caregiver reports, screening tool. | Rapid, easy to administer screening tool. | Does not assess all domains of neurodevelopment, contains certain culture-specific items, subject to reporting & recall bias. |
| Child Behavior Checklist (CBCL)^[[3]](#footnote-3)^ | Internalizing, externalizing, and total  problems | 1.5-5 years | 15-20 | Parent/caregiver reports, screening tool. | Easy to administer, short duration of assessment, well-established screening tool. | Subject to reporting & recall bias, assesses only one dimension of neurodevelopment, i.e. behavior. |
| Kilifi Developmental Inventory (KDI)^[[4]](#footnote-4)^ | (1) Locomotor skills (2) Eye-hand co-ordination. These are combined to create a psychomotor scale. | 0.5-3 years | 60 | Observer report, screening tool. | Range of neurodevelopmental outcomes from mild to severe assessed, easy to use, culturally appropriate for low resource settings, easy to score. | Long administration time, requires training lasting 1-2 months; domains of language & social emotional not assessed. |
| MacArthur-Bates Communicative Development inventory^[[5]](#footnote-5)^^[[6]](#footnote-6)^^[[7]](#footnote-7)^ | Language - receptive and expressive | 0.75 – 2.5 years | 20-40 | Parent report, screening tool. | Yields high outputs on word generation, comprehension & expression. Available in Spanish. | Only assess one construct i.e. language, subject to reporter & recall bias. |
| The Malawi Developmental Assessment Tool (MDAT)^[[8]](#footnote-8)^ | Gross motor, fine motor, language, social | 0-6 years | 30 | Observer report, screening tool. | Easy to use, short duration of administration, administered by midwives, culturally appropriate for low resource settings; good validity & reliability. | Assesses neurodevelopmental delay, not a well-established tool. |
| Mullen’s Scales of Early Learning^[[9]](#footnote-9)^ | Five scales: Gross Motor, Visual Reception, Fine Motor, Expressive Language, and Receptive Language. | Birth – 5.5 years | 15-30 | Observer rated, screening tool. | Short administration time, standardized, easy profile analysis, generates an early learning composite. | Modest correlations with the BSID have led to a lack of consensus on validity of the measure. |
| Rapid Neurodevelopmental Assessment (RNDA)^[[10]](#footnote-10)^ | Gross motor, fine motor, vision, hearing, speech, cognition, behavior and seizures. | 0-5 years | 30 | Observer report, screening tool. | Range of outcomes from mild to severe assessed, easy to use, culturally appropriate for low resource settings, validated against the BSID – good reliability & validity, short administration time. | Domains of motor development and language assessed by a single item, not widely used, relatively new tool. |
| The Ten Questions Screen^[[11]](#footnote-11)^ | Cognition, motor and seizures (serious neurodevelopmental delay). | 2-9 years | 10 | Maternal report, screening test. | Validated in 3 LMICs, large validation sample, and good specificity. | Assesses severe delays, performs differently in different populations; behavior, vision & hearing/auditory processing not assessed. |
| Diagnostic Instruments | | | | | | |
| Bayley Scales of Infant Development (BSID)^[[12]](#footnote-12)^ | Mental, physical and behavior development. | 0-3 years | 90 | Observer rated, diagnostic tool. | Widely used, good predictive and discriminant validity. | Specialized training, long administration time, difficult to administer in low resource settings, both items and norms are culturally biased. |
| Battelle Developmental Inventory^[[13]](#footnote-13)^ | Five domains: Cognitive, Motor, Adaptive, Personal-Social & communication (Language). | Birth-8 years | 30 | Parent report, observer report or mixed, diagnostic tool. | Subdomain scores, domain quotient, percentiles generated; Spanish version available. | Long administration time, needs specialist training, subject to reporting & recall bias. |
| Merrill Palmer revised scales^[[14]](#footnote-14)^ | Cognitive, language, motor & self-help domains | 0-6 years | 50 | Observer rated, diagnostic tool. | Yields standard scores, percentiles, developmental index; available in Spanish. | Lengthy to administer, requires specialist training. |
| The Griffith Mental Development Scales^[[15]](#footnote-15)^ | Locomotor, personal-social, hearing & language, eye-hand co-ordination, practical reasoning, performance. | 0-8 years | 50-60 | Observer rated, diagnostic test. | Well-established; good validity, yields accumulated scores, general quotient & developmental quotients. | Long duration of administration, requires specialist training. |

Table S5 Selection criteria for vision tests.

| **Essential Criteria:** |
| --- |
| 1. The test must be suitable to assess vision in 2 year olds. |
| 2. The test must assess the entire visual pathway and not merely specific components of the ophthalmic apparatus. |
| 3. The test must be sensitive enough to detect subtle differences in vision in a healthy cohort of children. |
| 4. The test must yield an objective score of vision. |
| 5. The test must possess established validity |
| 6. The measurement of vision must not be affected by disturbances in language development, cognition, and/or hearing. |
| 7. The test must be suitable for use in the developing world and in low-resource settings, and should not contain items that are culture or language specific. |
| 8. The duration of assessment for each individual child must not exceed 10 minutes. |
| 9. It must be easy to train local field workers to administer the test and no specialist training in ophthalmology, pediatrics or related disciplines must be necessary. |
| **Desirable Criteria:** |
| 1. The test should require minimal infrastructure and any equipment to administer the test must not be expensive. |
| 2. The test should assess more than one aspect of vision. |

Table S6 Selection criteria for neuropsychological tests (cognition, language, motor skills, behavior, attention and emotional reactivity).

| **Essential Criteria:** |
| --- |
| 1. The test must be suitable to assess neuropsychological function in 2 year olds. |
| 2. The test must assess the following aspects of neuropsychological function: cognition, language, motor skills, behavior, attention and emotional reactivity. |
| 3. The test must be sensitive enough to detect subtle differences in neurodevelopment in a healthy cohort of children. |
| 4. The test must characterize outcomes across a spectrum. |
| 5. The test must possess established reliability and validity in international settings. |
| 6. The test must be free from culture-specific items and suitable for use in the western and developing world, in both high and low income settings. |
| 7. The test must be based upon objective reporting and not subjective judgment of the child’s performance. |
| 8. The duration of assessment for each individual child must not exceed 30 minutes. |
| **Desirable Criteria:** |
| 1. The test should employ a combination of methodologies including but not limited to direct tests, observation and caregiver reports. |
| 2. The test should require minimal infrastructure and any equipment to administer the test must not be expensive. |

Table S7 Selection criteria for auditory function tests.

| **Essential Criteria:** |
| --- |
| 1. The test must be suitable for assessing auditory function in children at 2 years of age. |
| 2. The test must assess the entire auditory pathway and not merely specific components of the auditory apparatus. |
| 3. The test must be sensitive enough to detect subtle differences in auditory function in a healthy cohort of children. |
| 4. The technique must possess established validity and be a well-established measure of auditory function in children. |
| 5. The measurement of auditory function must not be affected by simultaneous disturbances in cognition, and/or vision. |
| 6. The technique must be suitable for use in the developing world and in low-resource settings, and should not contain items that are culture or language specific. |
| 7. The duration of assessment for each individual child should not exceed 20 mins. |
| 8. It must be easy to train local field workers to administer the test and no specialist training in ophthalmology, pediatrics or related disciplines should be necessary. |
| **Desirable Criteria:** |
| 1. Any equipment to administer the technique must not be expensive or difficult to use. |
| 2. The technique should yield objective measures of amplitude and latency of responses. |

Table S8 Selection criteria for sleep tests.

| **Essential Criteria:** |
| --- |
| 1. The test must be suitable for assessing sleep in children at 2 years of age. |
| 2. The test must be sensitive enough to detect subtle differences in sleep in a healthy cohort of children. |
| 3. The test must possess established validity and be a well-established measure of sleep in children. |
| 4. The test must be an objective measure of sleep in children. |
| 5. The test must be as non-disruptive to daily life as possible for both the child and the caregivers, and should not require prolonged periods of recording in laboratories. |
| 6. The test must measure sleep in a natural, home based setting. |
| 7. The test must be suitable for use in the developing world and in low-resource settings. |
| 8. It must be easy to train local field workers to administer the tool and no specialist training in pediatrics, child psychiatry, neuroscience or related disciplines must be necessary. |
| **Desirable Criteria:** |
| 1. Any equipment to administer the technique should not be expensive. |
| 2. The technique should yield information on a range of sleep characteristics including but not limited to sleep efficiency, total duration of sleep and night time awakenings. |
| 3. The technique should yield information about the extent of the child’s motor activity during the day. |

1. Bricker D, Squires J (1999) Ages and Stages Questionnaire: A Parent-Completed, Child-Monitoring System. 2 ed: Paul H. Brookes Publishing Co. [↑](#footnote-ref-1)
2. Briggs-Gowan MJ, Carter AS (2007) Applying the Infant-Toddler Social & Emotional Assessment (ITSEA) and Brief-ITSEA in early intervention. Infant Mental Health Journal 28: 564-583. [↑](#footnote-ref-2)
3. Koot H, Van Den Oord ECG, Verhulst F, Boomsma D (1997) Behavioral and Emotional Problems in Young Preschoolers: Cross-Cultural Testing of the Validity of the Child Behavior Checklist/2-3. Journal of Abnormal Child Psychology 25: 183-196. [↑](#footnote-ref-3)
4. Abubakar A, Holding P, van Baar A, Newton CRJC, van de Vijver FJR (2008b) Monitoring psychomotor development in a resourcelimited setting: an evaluation of the Kilifi Developmental Inventory Annals of Tropical Paediatrics: International Child Health 28: 217-226. [↑](#footnote-ref-4)
5. Charman T, Drew A, Baird C, Baird G (2003) Measuring early language development in preschool children with autism spectrum disorder using the MacArthur Communicative Development Inventory (Infant Form). Journal of Child Language 30: 213-236. [↑](#footnote-ref-5)
6. Fenson L, Dale PS, Reznick JS, Thal D, Bates E, et al. (1993) The MacArthur Communicative Development Inventories: User’s Guide and Technical Manual; H. P, editor. Baltimore: Brokes Publishing Co. [↑](#footnote-ref-6)
7. Fenson L, Dale P, Reznick J, Bates E, Thal D, et al. (1994) Variability in early communicative development. Monographs of the Society for Research in Child Development, Serial No 242, 59. [↑](#footnote-ref-7)
8. Gladstone MJ, Lancaster GA, Umar E, Nyirenda M, Kayira E, et al. (2010) The Malawi Developmental Assessment Tool (MDAT): The Creation, Validation, and Reliability of a Tool to Assess Child Development in Rural African Settings. PLoS Medicine. [↑](#footnote-ref-8)
9. Mullen EM (1995) Mullen Scales of Early Learning Circle Pines, MN: American Guidance Service Inc. [↑](#footnote-ref-9)
10. Khan NZ, Muslima H, Begum D, Shilpi AB, Akhter S, et al. (2010) Validation of Rapid Neurodevelopmental Assessment Instrument for Under-Two-Year-Old Children in Bangladesh. Pediatrics 125: e755-e762. [↑](#footnote-ref-10)
11. Durkin MS, Davidson LL, Desai P, Hasan ZM, Khan N, et al. (1994) Validity of the Ten Questions Screen for Childhood Disability: Results from Population-Based Studies in Bangladesh, Jamaica, and Pakistan. Epidemiology 5: 283-289. [↑](#footnote-ref-11)
12. Bayley N (2006) Bayley Scales of Infant Development Manual. Antonio, Texas: The Psychological Corporation. [↑](#footnote-ref-12)
13. Guidubaldi J, Perry JD (1984) Concurrent and Predictive Validity of the Battelle Development Inventory at the First Grade Level. Educational and Psychological Measurement 44: 977-985. [↑](#footnote-ref-13)
14. Roid GH, Sampers JL (2004) Merrill-Palmer-revised scales of development: Stoelting. [↑](#footnote-ref-14)
15. Griffiths R (1954) The abilities of babies: a study in mental measurement. New York, NY, US: McGraw-Hill. [↑](#footnote-ref-15)
